# Large differences in the organization of palliative care in nursing homes in six European countries: findings from the PACE cross-sectional study

**DOI:** 10.1186/s12904-021-00827-x

**Published:** 2021-08-25

**Authors:** E. Honinx, L. Van den Block, R. Piers, B. D. Onwuteaka-Philipsen, S. Payne, K. Szczerbińska, G. Gambassi, M. Kylänen, L. Deliens, T. Smets, on behalf of PACE, Yuliana Gatsolaeva, Rose Miranda, Lara Pivodic, Marc Tanghe, Hein van Hout, Roeline H. R. W. Pasman, Mariska Oosterveld-Vlug, Ruth Piers, Anne B. Wichmann, Yvonne Engels, Myrra Vernooij-Dassen, Jo Hockley, Sheila Payne, Suvi Leppäaho, Ilona Barańska, Sophie Pautex, Catherine Bassal, Federica Mammarella, Martina Mercuri, Paola Rossi, Ivan Segat, Agata Stodolska, Eddy Adang, Paula Andreasen, Outi Kuitunen-Kaija, Danni Collingridge Moore, Agnieszka Pac, Violetta Kijowska, Maud ten Koppel, Jenny T. van der Steen, Emilie Morgan de Paula

**Affiliations:** 1 End-of-Life Care Research Group, Department of Family Medicine & Chronic Care, Vrije Universiteit Brussel (VUB) & Ghent University, Laarbeeklaan 103, 1090 Brussels, Belgium; 2 Department of Geriatric Medicine, Ghent University Hospital, De Pintelaan 185, 9000 Ghent, Belgium; 3 EMGO Institute for Health and Care Research, Department of Public and Occupational Health, Expertise Center for Palliative Care, VU University Medical Center, Van der Boechorstraat 7, 1081 BT Amsterdam, The Netherlands; 4 Faculty of Health And Medicine, Lancaster University, 46 Bardsea, Bailrigg, Lancaster, LA14YX UK; 5 Department of Sociology of Medicine, Chair of Epidemiology and Preventive Medicine, Medical Faculty, Jagiellonian University Medical College, ul. Kopernika 7a, 31-034 Kraków, Poland; 6 Department of Internal Medicine, Istituto Di Medicina Interna E Geriatria, Università Cattolica del Sacro Cuore, Largo F. Vito, 1 – 00135 Rome, Italy; 7 National Institute for Health and Welfare, Mannerheimintie 166, P.O. Box 30, 00271 Helsinki, Finland

**Keywords:** Organization, Structural indicators, Palliative care, Nursing home, Europe, PACE

## Abstract

**Background:**

To be able to provide high-quality palliative care, there need to be a number of organizational structures available in the nursing homes. It is unclear to what extent such structures are actually present in nursing homes in Europe. We aim to examine structural indicators for quality of palliative care in nursing homes in Europe and to evaluate the differences in terms of availability of and access to palliative care, infrastructure for residents and families, multidisciplinary meetings and quality improvement initiatives.

**Methods:**

A PACE cross-sectional study (2015) of nursing homes in Belgium, England, Finland, Italy, the Netherlands and Poland. Nursing homes (*N* = 322) were selected in each country via proportional stratified random sampling. Nursing home administrators (*N* = 305) filled in structured questionnaires on nursing home characteristics. Organization of palliative care was measured using 13 of the previously defined IMPACT structural indicators for quality of palliative care covering four domains: availability of and access to palliative care, infrastructure for residents and families, multidisciplinary meetings and quality improvement initiatives. We calculated structural indicator scores for each country and computed differences in indicator scores between the six countries. Pearson’s Chi-square test was used to compute the p-value of each difference.

**Results:**

The availability of specialist palliative care teams in nursing homes was limited (6.1–48.7%). In Finland, Poland and Italy, specialist advice was also less often available (35.6–46.9%). Up to 49% of the nursing homes did not provide a dedicated contact person who maintained regular contact with the resident and relatives. The 24/7 availability of opioids for all nursing home residents was low in Poland (37.5%).

**Conclusions:**

This study found a large heterogeneity between countries in the organization of palliative care in nursing homes, although a common challenge is ensuring sufficient structural access to specialist palliative care services. Policymakers and health and palliative care organizations can use these structural indicators to identify areas for improvement in the organization of palliative care.

What is known on this topic:

- To be able to provide high-quality palliative care, there need to be a number of organizational structures available in the nursing homes
- It is unclear to what extent such structures are actually present in nursing homes in Europe.
- Gaining insight into these structures, via the IMPACT structural indicators for quality of palliative care, would help policymakers in identifying areas for improvement and in developing policy measures.

What this study adds:

- European nursing homes lack dedicated palliative care functions, specialist palliative care teams, and a contact person who maintains regular contact with the resident and relatives.
- The availability of opioids is low in nursing homes in Poland.
- Policymakers should invest in the availability of adequate general and specialist palliative care for all nursing home residents in need of this kind of support, either via internal or external services, with attention to minimal equipment and the necessary financial resources.

## Background

Delivering high-quality palliative care is of utmost importance in nursing homes given the complex needs of the residents living and dying in these facilities. Information about the quality of care can be drawn from care process and outcomes, but also from structure or organizational characteristics [1]. A recent EU study, the European IMPACT project (IMplementation of quality indicators for PAlliative Care sTudy) [2] developed structural indicators for quality of palliative care. The IMPACT structural indicators were developed for several palliative care settings including nursing homes and cover different domains such as access to specialist palliative care, availability of specialized equipment for residents in need of palliative care, multidisciplinary team meetings, availability of opioids and the assessment of experiences of the relatives with the care provided [2]. To be able to provide high-quality palliative care, there need to be a number of organizational structures available in the nursing homes [3].

To date, these structural indicators for quality of palliative care have not been used to measure quality in nursing homes, nor to compare countries via appropriate international samples, making it unclear to what extent such structures are actually present in nursing homes in Europe [4, 5]. Given the regulatory differences between nursing homes and between European countries, one can also expect differences in the way palliative care is organized in the nursing homes in these countries [6]. Insight into the organizational structures for palliative care in nursing homes in Europe will help policy and other decision-makers in identifying areas for improvement and in developing policy measures. To provide these insights, comparable data sets on structural indicators for quality of palliative care across different European countries are needed.

Within PACE, a European funded project that compared palliative care in nursing homes in six European countries, we conducted a large-scale survey of nursing homes to describe how they are organized with regard to palliative care, and to study differences in organizational structures [7]. The aim of the current study is to examine structural indicators for quality of palliative care in nursing homes in Belgium, England, Finland, Italy, the Netherlands and Poland and to evaluate the differences in terms of availability of and access to palliative care, infrastructure for residents and families, multidisciplinary meetings and quality improvement initiatives.

## Methods

### Study design

In 2015, we performed the PACE cross-sectional study of nursing homes in Belgium (Flanders), England, Finland, Italy, the Netherlands and Poland. Proportional stratified random sampling was used to select a representative sample of nursing homes in each country. Nursing homes were first stratified by region, then by nursing home type and bed capacity (higher or lower than the median number of beds in the country). They were sampled randomly and proportionally from each stratum. Where a nursing home declined to participate, another from the same stratum was sampled. For recruitment, national (or regional) lists of nursing homes were used, except for Italy, where no such list was available. In that country, a previously constructed cluster of nursing homes interested in research was used. To enhance recruitment of nursing homes in England, we relied on the ENRICH (Enabling Research In Care Homes) network [8]. The PACE protocol provides more details about the study [7].

### Setting and participants

We define nursing homes as ‘collective institutional settings where care, on-site provision of personal assistance with activities of daily living, and on-site or off-site provision of nursing and medical care, is provided for older people who live there, 24 h a day, 7 days a week, for an undefined period of time’ [9]. We distinguished three types of nursing homes: type 1 with 24/7 care from on-site physicians and nurses/care assistants, type 2 with 24/7 care from on-site nurses/care assistants and off-site physicians, and type 3 with 24/7 care from on-site care assistants, and off-site nurses and physicians [10]. The administrator of each participating nursing home was asked to fill in a structured questionnaire on nursing home characteristics.

### Data collection

A letter that introduced the PACE project was sent to the nursing home administrator inviting participation in the study. Upon agreement, a researcher visited the nursing home. During the visit, the administrator was asked to fill in a questionnaire about the nursing home.

### Measurements

The sample of nursing homes was described based on type of nursing home (care from on-site physicians and nurses / care from on-site nurses and off-site physicians / care from off-site physicians and nurses), type of ownership of the nursing home (public-nonprofit/private-nonprofit/private-profit) and size of nursing home (number of beds). The organization of palliative care in the nursing homes was measured using the IMPACT structural indicators for quality of palliative care [2]. The IMPACT Indicators were previously developed in a European study to monitor and improve the organization of palliative care in a variety of settings [11]. The final set consists of 23 indicators, covering seven organizational domains: 1) access to palliative care, 2) infrastructure, 3) assessment tools, 4) personnel, 5) documentation of clinical data, 6) quality, and 7) education. From this set, 13 indicators, covering four domains (1, 2, 4 and 6), are applicable to nursing homes and were used in the PACE questionnaire for nursing home administrators. These indicators were included in a feasibility testing of the questionnaires in several nursing homes in each country. Based on the feedback of the feasibility testing, the PACE consortium made minor adjustments to the wording of the domains and indicators that required clarification. The final four domains were: availability of and access to palliative care, infrastructure for residents and families, multidisciplinary meetings and quality improvement initiatives. The original thirteen IMPACT indicators and the adapted thirteen structural indicators for quality of palliative care can be found in Table 1. The indicators were measured in all six countries. However, the availability of in-house and external palliative care services in a nursing home varies between countries. To properly interpret the scores on the indicators for availability of palliative care, one must have an understanding of what services actually exist in the different countries. Table 2 gives an overview of the palliative care services available to nursing homes in the six countries, which can be used as a framework for the interpretation of the indicators within the specific context of each country.

**Table 1:** Overview of the wording of the original IMPACT structural indicators for palliative care and adjustments made in the PACE questionnaire

**IMPACT indicators**	**PACE indicators**
***1. Access to palliative care***	***1. Availability of and access to palliative care***
1. A **specialist palliative care team** is available 24/7.	Is there a **specialist palliative care team** present in your facility (employed in your facility)? If yes, do you **use this specialist palliative** care team?
2. **Specialist palliative care** advice is available 24/7 to professionals delivering palliative care.	Is **specialist palliative care advice** available to professionals delivering palliative care in your facility? If yes, do you **use this advice**?
3. Bereaved relatives and/or professionals involved in care of a person in need of palliative care are offered support during the **bereavement** process if they need or wish to have support.	Do you have a procedure in place to ensure that relatives of residents are offered **bereavement support**, if they need or wish to have support?
4. **Opioids** are accessible and available for persons in need of palliative care 24/7.	Are **opioids** available 24/7 for residents in need of palliative care in your facility?
5. Persons in need of palliative care have an assigned **contact person** who maintains regular contact with the person and their families and ensures coordinated delivery of health and social care.	Does your facility offer residents in need of palliative care an **assigned contact person** (e.g. care manager, case manager or key worker) who maintains a regular contact with the resident and his or her relatives, in order to ensure coordinated health and social care?
***2. Infrastructure***	***2. Infrastructure for residents and families***
6. **Specialized equipment** (e.g. anti-decubitus mattresses, suction equipment, stoma care, oxygen delivery, drug administration pumps, hospital beds, etc.) is available to persons in need of palliative care.	What **specialized equipment** is available for residents in need of palliative care in your facility? (pressure relieving mattresses, suction equipment, stoma care supplies, oxygen delivery, syringe driver, hospital beds)
7. **Single bedrooms** are available for persons who are dying and who wish to have one.	Are **single bedrooms** available in your facility for ALL residents who are dying and who wish to have one?
8. Family members and friends are able to **visit** the dying person without restrictions of visiting hours.	Are there unrestricted **visiting hours** for relatives of residents who are dying, if they wish?
9. There are facilities for relatives to **stay overnight** with their dying relative.	Are there facilities for relatives to **stay overnight** with their dying relative?
***3. Personnel***	***3. Multidisciplinary meetings***
10. The multidisciplinary **team** that delivers palliative care services consists of at least: a) a physician and nurse;	Is there a **regular multidisciplinary meeting** (with at least a physician and a nurse) to review treatment and care plans organized in your facility?
11. There is a **weekly multidisciplinary meeting** with at least the physician and nurse in charge of the person in need of palliative care to review treatment and care plans.	If yes, **how frequently** is this meeting organized? (**weekly**, monthly, other frequency)
***4. Quality***	***4. Quality improvement initiatives***
12. **Family and caregiver experiences** of the palliative care service are assessed/evaluated/recorded.	Does your facility systematically **assess the experiences of relatives** of residents regarding provided care?
13. An end-of-life care **pathway** (such as the Liverpool Care Pathway) was used for the **last 3 days of life** of a person in need of palliative care.	Are **specific guidelines** used for the last 3 days of life of a resident in need of palliative care in your facility?

**Table 2:** Classification of in-house and external palliative care services available in nursing homes in six countries

**Belgium**	**Netherlands**	**England**	**Finland**	**Italy**	**Poland**
**In-house palliative care service in a nursing home**					
NH staff (nurse/care assistants) trained in palliative care Palliative care support team per facility, consisting of: - Coordinating advisory physician (a GP who is responsible for nunrsing home medical management incl. palliative care; however, s/he does not function as a GP of the residents (they have their own GP) -Palliative care reference nurse (to support GPs and nurses operating in the nursing homes)	Type 1: Elderly care physician (also provides general palliative care) and nurse/care assistants trained in palliative care Type 2: Nurse/care assistants trained in palliative care	NH staff (nurse/care assistants) trained in palliative care Palliative care team (specialist nurse and palliative medicine doctor, sometimes other professionals) advise and assess residents, no hands-on care. Some palliative care nurses are linked to the nursing home	Elderly care physicians and registered and licensed practical nurses trained in palliative care	General practitioner and NH staff (nurse/care assistants) trained in palliative care	Type 1: General practitioner and NH staff (nurse/care assistants) trained in palliative care Type 2: no internal palliative care service Difference in availability specialist palliative care advice between type 1 and 2 because of financial contracting law regulation Type 1 is obliged to provide palliative care in the frame of internal service (call on palliative care specialist is an exception). Type 2 calls on external palliative care specialists
**External palliative care service in a nursing home**					
External palliative home care team (provides advice or training to nursing home staff) External general physician	Type 1: Access to consultant or consultation team palliative care Type 2: External elderly care physician (also provides general palliative care) Access to consultant or consultation team palliative care	External physicians linked to the nursing home District nurses (deliver hands-on care to older people and provide general palliative care) sometimes linked to the nursing home External specialist palliative care team and care home liaison specialist palliative care nurse	External general physician External elderly care physician or external palliative care team	Palliative care services of the health care district in isolated cases Palliative care services from collaborating not-for-profit organizations	Type 1: no use of external palliative care service Type 2: Hospice team (palliative care specialist physician and palliative care specialist nurse) visiting a resident at residential home

### Statistical analysis

All analyses were performed with IBM SPSS version 23 [12]. For each country, the types and mean size of nursing homes and types of nursing home ownership were determined. Then, we calculated indicator scores for each country, based on the structural indicators in the nursing homes, reported as counts and percentages. Next, we computed differences in indicator scores between the six countries. Pearson’s Chi-square test was used to compute the p-value of each difference; Fisher’s exact test was applied for frequencies below 5. An alpha level of *p* < 0.05 defined statistical significance. Because all countries, except Belgium and Finland, had several types of nursing homes, we additionally calculated indicator scores by nursing home type within those four countries. Due to convergence problems because of low numbers of cases, the significance of these differences could not be tested.

## Results

For this study, 322 nursing homes from Belgium, England, Finland, Italy, the Netherlands and Poland were initially recruited. Of these 322 homes, a total of 305 (95%) nursing homes – for which the nursing home administrator returned the questionnaire on nursing home characteristics – were eventually included.

### Characteristics of the participating nursing homes

Table 3 shows that type 1 nursing homes (care from on-site physicians and nurses) existed in Italy, the Netherlands and Poland (24.2–38.6%). Type 2 nursing homes (care from on-site nurses and off-site physicians) were the most common type in all countries (55.1–100%) except in England (45.8%), where type 3 nursing homes (care from off-site physicians and nurses) were most common (54.2%; *p* < 0.001). In almost all countries, the majority of the nursing homes were public non-profit facilities, except for England and Italy where more private for-profit facilities (86.8 and 41.8% respectively) existed (*p* < 0.001). Nursing home size differed significantly between a mean of 37 beds in Finland to 110 beds in Belgium (*p* < 0.001).

**Table 3:** Description of sample of nursing homes in six European countries (*N* = 305)

	**BE*** N* = 43	**NL*** N* = 44	**EN*** N* = 48	**FI*** N* = 88	**IT*** N* = 33	**PL*** N* = 49	*P*-value
	N(%)	N(%)	N(%)	N(%)	N(%)	N(%)	
**Type of nursing home**							** < 0.001**
Type 1 with on-site care from physicians, nurses and care assistants	NA	17 (38.6)	NA	NA	8 (24.2)	22 (44.9)	
Type 2 with on-site care from nurses and care assistants, but off-site care from physicians	43 (100.0)	27 (61.4)	22 (45.8)	88 (100.0)	25 (75.8)	27 (55.1)	
Type 3 with on-site care from care assistants, but off-site care from nurses and physicians	NA	NA	26 (54.2)	NA	NA	NA	
**Type of ownership of nursing home**							** < 0.001**
Public-nonprofit	21 (48.8)	44 (100.0)	1 (2.1)	66 (75.9)	10 (30.3)	30 (62.5)	
Private-nonprofit	19 (44.2)	NA	5 (10.4)	10 (11.5)	8 (24.2)	17 (35.4)	
Private-profit	3 (7.0)	NA	42 (87.5)	11 (12.6)	15 (45.5)	1 (2.1)	
**Size of nursing home**							** < 0.001**
Mean (SD) number of beds	110 (49)	88 (54)	39 (22)	37 (28)	82 (56)	72 (41)	

Missing values: Type of nursing home = 0, type of ownership = 2, size = 5

### Availability of and access to palliative care in nursing homes

Specialist palliative care teams employed by the nursing home were rarely present, with a prevalence rate ranging from 6.1% in England up to 48.7% in Belgium (*p* < 0.001; Table 4). Specialist palliative care advice for professionals delivering palliative care in the nursing home was mainly available in Belgium (92.7%), England (87.2%) and the Netherlands (65.1%). In the other countries, specialist advice was less often available (35.6% in Finland—46.9% in Italy; *p* < 0.001). The majority of the nursing homes in England, Finland and Italy had a procedure in place regarding bereavement support for relatives (respectively 68.2, 59.8 and 59.4%); whereas in Belgium (26.2%), the Netherlands (22%) and Poland (20.4%) this was the case to a lesser extent (*p* < 0.001). Opioids were available 24/7 to all residents in the majority of the nursing homes in all countries (69.7% in Italy – 83.3% in England), except for Poland, where they were available 24/7 to all residents in only 37.7% of the nursing homes (*p* < 0.001). In most nursing homes an assigned contact person who maintains a regular contact with the resident and relatives was available for each resident (51.2% in Belgium – 82.6% in England; *p* < 0.001), except for Poland and Italy where such a contact person was often not present at all (not available: Poland 57.1%; Italy 48.5%). When comparing indicator scores of the different nursing home types (Table 5 in Appendix), a large difference was found in Poland regarding the availability of specialist palliative care advice for professionals delivering palliative care between type 1 homes (care from on-site physicians and nurses) and type 2 homes (care from on-site nurses and off-site physicians) (25 and 61.5% respectively).

**Table 4:** Structural indicators in nursing homes in six European countries (*N* = 305)

	**BE*** N* = 43	**NL*** N* = 44	**EN*** N* = 48	**FI*** N* = 88	**IT*** N* = 33	**PL*** N* = 49	*P*-value*
	N(%)	N(%)	N(%)	N(%)	N(%)	N(%)	
**Availability of specialist palliative care**							
**Specialist PC team present in the facility (employed by facility)?**							
Yes	19 (48.7)	13 (29.5)	3 (6.5)	8 (9.5)	4 (12.1)	3 (6.1)	** < 0.001**
**If present, use specialist PC team?**							
Yes, for all residents in need of SPC	11 (61.1)	1 (7.7)	2 (66.7)	4 (50.0)	2 (66.7)	2 (66.7)	** < 0.05**
Yes, for most residents in need of SPC	3 (16.7)	1 (7.7)	0 (0.0)	0 (0.0)	1 (33.3)	0 (0.0)	
Yes, for some residents in need of SPC	4 (22.2)	7 (53.8)	1 (33.3)	4 (50.0)	0 (0.0)	1 (33.3)	
No, never	0 (0.0)	4 (30.8)	0 (0.0)	0 (0.0)	0 (0.0)	0 (0.0)	
**Specialist PC advice available to professionals delivering PC in your facility?**							
Yes	38 (92.7)	28 (65.1)	41 (87.2)	31 (35.6)	15 (46.9)	21 (45.7)	** < 0.001**
**If available, use specialist PC advice?**							
Yes, for all residents in need of SPC	14 (38.9)	5 (19.2)	30 (81.1)	11 (40.7)	8 (57.1)	11 (57.9)	** < 0.001**
Yes, for most residents in need of SPC	6 (16.7)	2 (7.7)	2 (5.4)	3 (11.1)	1 (7.1)	0 (0.0)	
Yes, for some residents in need of SPC	16 (44.4)	15 (57.7)	5 (13.5)	10(37.0)	4 (28.6)	5 (26.3)	
No, never	0 (0.0)	4 (15.4)	0 (0.0)	3 (11.1)	1 (7.1)	3 (15.8)	
**Procedure bereavement support relatives?**							
Yes	11 (26.2)	9 (22.0)	30 (68.2)	52 (59.8)	19 (59.4)	10 (20.4)	** < 0.001**
**Opioids available 24/7 for residents with PC?**							
Yes, for all residents	33 (78.6)	32 (72.7)	35 (83.3)	70 (80.5)	23 (69.7)	18 (37.5)	** < 0.001**
Yes, for most residents	1 (2.4)	4 (9.1)	4 (9.5)	6 (6.9)	3 (9.1)	1 (2.1)	
Yes, for some residents	7 (16.7)	4 (9.1)	2 (4.8)	7 (8.0)	4 (12.1)	16 (33.3)	
No, never	1 (2.4)	4 (9.1)	1 (2.4)	4 (4.6)	3 (9.1)	13 (27.1)	
**Contact person for residents/relatives to ensure coordinated health and social care?**							
Yes, for all residents	21 (51.2)	32 (72.7)	38 (82.6)	63 (71.6)	15 (45.5)	19 (38.8)	** < 0.001**
Yes, for most residents	5 (12.2)	1 (2.3)	2 (4.3)	5 (5.7)	0 (0.0)	0 (0.0)	
Yes, for some residents	4 (9.8)	0 (0.0)	4 (8.7)	3 (3.4)	2 (6.1)	2 (4.1)	
No, never	11 (26.8)	11 (25.0)	2 (4.3)	17 (19.3)	16 (48.5)	28 (57.1)	
**Facilities for residents and families**							
**Specialized equipment available**^**a**^							
Pressure relieving mattresses	42 (97.7)	43 (97.7)	46 (95.8)	80 (90.9)	33 (100.0)	47 (95.9)	0.377
Suction equipment	34 (79.1)	34 (77.3)	19 (39.6)	70 (79.5)	33 (100.0)	45 (91.8)	** < 0.001**
Stoma care supplies	29 (67.4)	38 (86.4)	24 (50.0)	67 (76.1)	25 (75.8)	35 (71.4)	** < 0.01**
Oxygen delivery	42 (97.7)	43 (97.7)	32 (66.7)	61 (69.3)	30 (90.9)	34 (69.4)	** < 0.001**
Syringe driver	28 (65.1)	32 (72.7)	40 (83.8)	34 (38.6)	12 (36.4)	21 (42.9)	** < 0.001**
Hospital beds	37 (86.0)	40 (90.9)	43 (89.6)	87 (98.9)	26 (78.8)	47 (95.9)	** < 0.01**
**Single bedrooms in facility for ALL dying residents who wish to have one?**							
Yes	34 (79.1)	40 (93.0)	43 (93.5)	69 (78.4)	16 (50.0)	13 (27.1)	** < 0.001**
**Unrestricted visiting hours for relatives of dying residents if they wish?**							
Yes	43 (100.0)	40 (93.0)	40 (87.0)	88 (100.0)	31 (96.9)	47 (95.9)	** < 0.01**
**Relatives can stay overnight with dying relative**							
Yes	36 (87.8)	38 (88.4)	36 (90.0)	72 (81.8)	30 (90.9)	29 (59.2)	**0.001**
**Multidisciplinary approaches**							
**Regular multidisciplinary meeting (with at least a physician and a nurse) to review treatment and care plans organized in your facility?**							
Yes	37 (88.1)	43 (97.7)	29 (64.4)	44 (50.6)	26 (78.8)	35 (72.9)	** < 0.001**
**If yes, how frequently is this meeting organized?**							
Weekly	8 (21.6)	12 (27.9)	11 (37.9)	8 (18.2)	13 (50)	12 (34.3)	** < 0.01**
**Quality improvement initiatives**							
**Systematic assessment of relatives’ experiences regarding provided care?**							
Yes, for all residents	18 (45.0)	18 (40.9)	29 (64.4)	29 (33.7)	20 (62.5)	5 (10.2)	** < 0.001**
Yes, for most residents	7 (17.5)	4 (9.1)	5 (11.1)	21 (24.4)	1 (3.1)	7 (14.3)	
Yes, for some residents	6 (15.0)	6 (13.6)	2 (4.4)	25 (29.1)	4 (12.5)	5 (10.2)	
No, never	9 (22.5)	16 (36.4)	9 (20.0)	11 (12.8)	7 (21.9)	32 (65.3)	
**Specific guidelines used for the last 3 days of life of a resident in need of palliative care in your facility?**							
Yes	15 (36.6)	18 (40.9)	21 (51.2)	36 (40.9)	3 (9.7)	7 (14.9)	** < 0.001**

Missing values: Specialist PC team = 10, use specialist PC team = 2 (255 not applicable because no specialist PC team present), specialist PC advice = 9, use specialist PC advice = 24 (122 not applicable because no specialist PC advice available), procedure bereavement = 10, opioids = 9, contact person = 4, specialized equipment = 0, single bedrooms = 5, unrestricted visiting hours = 4, relatives overnight = 11, multidisciplinary meeting = 6, weekly multidisciplinary meeting = 15, systematic assessment relatives = 9, guidelines = 13

*Abbreviations*: *PC* Palliative Care, *SPC* Specialist Palliative Care

^*^χ2 test for differences between countries or Fisher’s exact test in case cells with frequency below 5, α = 0.05

^a^Multiple answers possible

### Infrastructure for residents and families in nursing homes

Pressure relieving matrasses (90.9% in Finland – 100% in Italy; *p* < 0.001), hospital beds (78.8% in Italy – 98.9% in Finland; *p* < 0.01) and oxygen delivery (66.7% in England – 97.7% in Belgium and the Netherlands; *p* < 0.001) were available in nearly all nursing homes. Suction equipment was present in nursing homes in all countries (77.3% in the Netherlands – 100% in Italy) but to a lesser extent in nursing homes in England (39.6%; *p* < 0.01). The same applied to stoma care supplies (50% in England – 86.4% in the Netherlands; *p* < 0.001). Syringe drivers were mainly available in nursing homes in England (83.8%), the Netherlands (72.7%) and Belgium (65.1%) but less frequently in the other countries (36.4% in Italy – 42.9% in Poland; *p* < 0.001). Additional analyses (Table 5 in Appendix) indicated a lesser supply of syringe drivers (3.7%) and oxygen delivery (48.1%) in type 2 homes (care from on-site nurses and off-site physicians) in Poland compared with type 1 homes (care from on-site physicians and nurses; 90.9 and 95.5% respectively). In England, suction equipment and stoma care supplies were less available in type 3 homes (care from off-site physicians and nurses; 11.5 and 30.8%) than in type 2 homes (72.7% for both indicators). In most countries, the nursing homes had single bedrooms available for dying residents who wished for one (78.4% in Finland – 93.5% in England; *p* < 0.001). This was usually not the case in Poland (27.1%) and Italy (50%). The large majority of nursing homes in the six countries permitted unrestricted visiting hours (87% in England – 100% in Belgium and Finland; *p* < 0.01) and overnight stays (59.2% in Poland – 90.9 in Italy; *p* = 0.001) for relatives of dying residents.

### Multidisciplinary meetings in nursing homes

Multidisciplinary meetings were held in the majority of nursing homes in all countries (50.6% in Finland – 97.7% in the Netherlands; *p* < 0.001) although the frequency of the meetings differed significantly between countries (9.4% in Italy – 39.4 in Finland; *p* < 0.01). There were no substantial differences between the different nursing home types within countries.

### Quality improvement initiatives in nursing homes

For 33.7–64.4% of the nursing homes, the experiences of the relatives of all residents regarding the care provided were assessed (*p* < 0.001). In Poland, this percentage was considerably lower (10.2%) with 65.3% of nursing homes never assessing relatives’ experiences. In the Netherlands, Italy and Poland, the assessment was carried out less often in type 2 nursing homes (care from on-site nurses and off-site physicians) than in type 1 homes (care from on-site physicians and nurses; Table 5 in Appendix). Guidelines for the last 3 days of life were often not used (48.8% in England – 90.3% in Italy; *p* < 0.001).

## Discussion

### Main findings

Using previously defined quality indicators for the organization of palliative care, this study identified several areas of improvement for the organization of palliative care in nursing homes in six European countries. Dedicated palliative care functions, specialist palliative care teams, and a dedicated contact person who maintained regular contact with the resident and relatives were often not structurally embedded in the organization of nursing home care in most countries, in particular in Finland and Poland. There was little structural availability of specialist advice for professionals delivering palliative care in Finland, Italy and Poland. The availability of opioids was low in nursing homes in Poland. Almost all structural indicators for quality of palliative care differed significantly between countries. Differences between nursing home types were limited to the availability of specialist palliative care advice, the regular assessment of relatives’ care experiences and the availability of certain specialized equipment.

### Strengths and limitations

This study has several strengths. It is the first study to measure and compare the structural indicators for quality of palliative care in nursing homes in representative samples of nursing homes that cover different European regions. Also, the overall response rate was very high (95%). We were able to include data from 305 nursing homes in six countries to make cross-country comparisons.

This study has some limitations. Firstly, we cannot exclude the risk of nursing homes with a strong interest in palliative care being more inclined to participate in the study. Secondly, although we were able to include representative samples of nursing homes for each country, the participating nursing homes only represent a small proportion of the total number of nursing homes in a country. Caution is thus needed when generalizing the results to other nursing homes. Third, we were unable to test the statistical significance of the differences in indicator scores between nursing home types within the countries due to low number of cases, but this is likely to account for some of the variance. Lastly, these structural indicators are mainly valuable for policymaking, to map the organization of palliative care and compare it on a macro-level. They do not guarantee high-quality palliative care for each individual resident, and a low score on an indicator does not necessarily indicate suboptimal palliative care for an individual resident. This was confirmed in a previous PACE study that found that even in countries with high levels of palliative care integration, the quality of dying and of end-of-life care of nursing home residents was poor [13].

### What this paper adds

Although we found heterogeneity in the way palliative care is organized in the different countries, one area that appears to need improvement in all countries is the availability and access to specialist palliative care support. We found that there is low access to specialist palliative care teams in all countries and there is little availability of specialist advice for professionals delivering palliative care in nursing homes in Finland, Italy and Poland. Belgium had the highest number of nursing homes indicating that a specialist palliative care team was present in the facility (49%) and that specialist advice (93%) was available. However, these results should be interpreted cautiously; in Belgium the number of employed staff is 0.5 FTE per resident which is not enough to provide adequate care to heavily care-dependent residents [14]. The availability and amount of specialist palliative care in a country also depends on the health care system of each country. However, although all nursing and care staff in nursing homes should be able to provide general palliative care to residents, research shows that a general palliative care approach is not always sufficient to meet the needs of certain nursing home residents; these residents may benefit from specialist palliative care, especially when needs become complex [15–17]. Our study shows that specialist palliative care is often not available in nursing homes, and there is thus clearly room for improvement in this area.

Aside from similarities, there were also apparent differences between countries, an important one being the low levels of access to opioids in nursing homes in Poland. The low opioid availability we found in nursing homes in Poland is not surprising as other research has shown that use and prescription of opioids was lowest in Poland compared with other European countries [18–21]. Another PACE study on opioid use at the end of life suggested that the low opioid prescription rate and use during the last days of life in nursing homes in Poland probably reflects the low opioid use in Poland in general [22, 23]. Opioids are important to treat pain at the end of life in nursing home residents and they are considered essential to providing adequate palliative care [16, 23, 24]. However, fear of opioid addiction, or analgesic tolerance, possible side-effects or the belief that opioids would hasten death might hinder access to and use of opioids [25, 26]. Our finding may be an incentive for policymakers in Poland to make opioids available 24/7 for all nursing home residents when they need them. To do this, the report of the Access To Opioid Medication in Europe (ATOME) group suggests that legal barriers that hinder the use of opioids in Poland need to be addressed, and that the misconception of opioids as being a life-shortening drug need to be corrected through adequate information and training for health care providers [27, 28].

Countries like Italy, Finland and Poland appear to have more areas for organizational improvement of palliative care (e.g. access to palliative care, availability of a contact person for residents or relatives and specialized equipment) than Belgium, England and the Netherlands. These results are in line with earlier findings from a report of the European Association of Palliative Care Taskforce on Long-term Care Facilities on the development of palliative care in nursing homes in Europe. In Poland, and especially in Italy and Finland, fewer initiatives to develop palliative care in nursing homes and less engagement by and within nursing homes with palliative care initiatives or care provision existed than in Belgium, the Netherlands, and the UK [6, 29]. Policy choices (e.g. regarding health care organization or funding) in a country affect the possibilities available to palliative care in that country [30]. Our findings confirm that the basic requirements for palliative care on a macro level are better guaranteed in Belgium, the Netherlands and the UK than in the other three countries. The differences in palliative care organization between countries may also reflect country-specific differences in culture, regulatory mechanisms and legislation [30, 31]. Specially in countries with room for improvement of palliative care organization, such as Italy, Poland and Finland, extra initiatives should be considered, while drawing lessons from other countries. Policymakers should invest in dedicated functions for palliative care and the availability of adequate general and specialist palliative care for all nursing home residents in need of this kind of support, either via internal or external services.

Nevertheless, the organization of palliative care in nursing homes remains a common challenge. This is due to the slow development of and limited attention given to palliative care in nursing homes [29]. Earlier research has shown that, regarding palliative care, focus was and still is mainly on hospitals, hospices and home care [32, 33]. Also with regard to general care and wellbeing, the nursing home setting remains underinvested [34]. The current study highlights the need for a model within the healthcare sector that guarantees equal access to palliative care for all healthcare settings, with detailed information on minimal equipment that should be available in nursing homes and the financial resources needed to provide it.

## Conclusions

This study found a large heterogeneity between countries in the organization of palliative care in nursing homes, although a common challenge is ensuring sufficient structural access to specialist palliative care services. Policymakers and health and palliative care organizations can use these structural indicators to identify areas for improvement in the organization of palliative care.

## Data Availability

The datasets used and/or analyzed during the current study are available from the corresponding author on reasonable request.

## Appendix

**Table 5:** Structural indicators in nursing homes by nursing home type within countries

By nursing home type^a^	**NL*** N* = 44		**EN*** N* = 48		**IT*** N* = 33		**PL*** N* = 49	
	N(%)		N(%)		N(%)		N(%)	
	Type 1	Type 2	Type 2	Type 3	Type 1	Type 2	Type 1	Type 2
	*N* = 17	*N* = 27	*N* = 22	*N* = 26	*N* = 8	*N* = 25	*N* = 22	*N* = 27
**Availability of specialist palliative care**								
**Specialist PC team present in the facility (employed by facility)?**								
Yes	6 (35.4)	7 (25.9)	0 (0)	3 (12)	0 (0)	4 (16)	2 (9.1)	1 (3.7)
**If present, use specialist PC team?**								
Yes, for all residents in need of SPC	1 (16.7)	0 (0)	N.A	2 (66.7)	N.A	2 (66.7)	1 (50)	1 (100)
Yes, for most residents in need of SPC	0 (0)	1 (14.3)	N.A	0 (0)	N.A	1 (33.3)	0 (0)	0 (0)
Yes, for some residents in need of SPC	2 (33.3)	5 (71.4)	N.A	1 (33.3)	N.A	0 (0)	1 (50)	0 (0)
No, never	3 (50)	1 (14.3)	N.A	0 (0)	N.A	0 (0)	0 (0)	0 (0)
**Specialist PC advice available to professionals delivering PC in your facility?**								
Yes	12 (70.6)	16 (61.5)	20 (90.9)	21 (84)	3 (37.5)	12 (50)	5 (25)	16 (61.5)
**If available, use specialist PC advice?**								
Yes, for all residents in need of SPC	4 (33.3)	1 (7.1)	14 (73.7)	16 (88.9)	1 (33.3)	7 (63.6)	2 (40)	9 (64.3)
Yes, for most residents in need of SPC	0 (0)	2 (14.3)	2 (10.5)	0 (0)	0 (0)	1 (9.1)	0 (0)	0 (0)
Yes, for some residents in need of SPC	4 (33.3)	11 (78.6)	3 (15.8)	2 (11.1)	1 (33.3)	3 (27.3)	2 (40)	3 (21.4)
No, never	4 (33.3)	0 (0)	0 (0)	0 (0)	1 (33.3)	0 (0)	1 (20)	2 (14.3)
**Procedure bereavement support relatives?**								
Yes	6 (35.3)	3 (12.5)	15 (71.4)	15 (65.2)	6 (75)	13 (54.2)	4 (18.2)	6 (22.2)
**Opioids available 24/7 for residents with PC?**								
Yes, for all residents	15 (88.2)	17 (63)	17 (81)	18 (85.7)	5 (62.5)	18 (72)	9 (40.9)	9 (34.6)
Yes, for most residents	2 (11.8)	2 (7.4)	3 (14.3)	1 (4.8)	1 (12.5)	2 (8)	1 (4.5)	0 (0)
Yes, for some residents	0 (0)	4 (14.8)	1 (4.8)	1 (4.8)	1 (12.5)	3 (12)	4 (18.2)	12 (46.2)
No, never	0 (0)	4 (14.8)	0 (0)	1 (4.8)	1 (12.5)	2 (8)	8 (36.4)	5 (19.2)
**Contact person for residents/relatives to ensure coordinated health and social care?**								
Yes, for all residents	15 (88.2)	17 (63)	17 (81)	21 (84)	3 (37.5)	12 (48)	7 (31.8)	12 (44.4)
Yes, for most residents	0 (0)	1 (3.7)	2 (9.5)	2 (8)	0 (0)	0 (0)	0 (0)	0 (0)
Yes, for some residents	0 (0)	0 (0)	2 (9.5)	2 (8)	1 (12.5)	1 (4)	2 (9.1)	0 (0)
No, never	2 (11.8)	9 (33.3)	0 (0)	2 (8)	4 (50)	12 (48)	13 (59.1)	15 (55.6)
**Facilities for residents and families**								
**Specialized equipment available†**								
Pressure relieving mattresses	17 (100)	26 (96.3)	21 (95.5)	25 (96.2)	8 (100)	25 (100)	21 (95.5)	26 (96.3)
Suction equipment	17 (100)	17 (63)	16 (72.7)	3 (11.5)	8 (100)	25 (100)	21 (95.5)	24 (88.9)
Stoma care supplies	15 (88.2)	23 (85.2)	16 (72.7)	8 (30.8)	4 (50)	21 (84)	16 (72.7)	19 (70.4)
Oxygen delivery	17 (100)	26 (96.3)	19 (86.4)	13 (50)	7 (87.5)	26 (96.3)	21 (95.5)	13 (48.1)
Syringe driver	13 (76.5)	19 (70.4)	20 (90.9)	20 (76.9)	2 (25)	10 (40)	20 (90.9)	1 (3.7)
Hospital beds	15 (88.2)	25 (92.6)	20 (90.9)	23 (88.5)	7 (87.5)	19 (76)	21 (95.5)	26 (96.3)
**Single bedrooms in facility for ALL dying residents who wish to have one?**								
Yes	16 (94.1)	24 (92.3)	19 (90.5)	24 (96)	4 (50)	12 (50)	6 (28.6)	7 (25.9)
**Unrestricted visiting hours for relatives of dying residents if they wish?**								
Yes	16 (94.1)	24 (92.3)	18 (85.7)	22 (88)	8 (100)	23 (95.8)	21 (95.5)	26 (96.3)
**Relatives can stay overnight with dying relative**								
Yes	17 (100)	21 (80.8)	17 (89.5)	19 (90.5)	7 (87.5)	23 (92)	13 (59.1)	16 (59.3)
**Multidisciplinary approaches**								
**Regular multidisciplinary meeting (with at least a physician and a nurse) to review treatment and care plans organized in your facility?**								
Yes	17 (100)	26 (96.3)	16 (76.2)	13 (54.2)	8 (100)	18 (72)	15 (68.2)	20 (76.9)
**If yes, how frequently is this meeting organized?**								
Weekly	3 (17.6)	9 (34.6)	5 (31.3)	6 (46.2)	4 (50)	9 (50)	5 (33.3)	7 (35)
**Quality improvement initiatives**								
**Systematic assessment of relatives’ experiences regarding provided care?**								
Yes, for all residents	8 (47.1)	10 (37)	11 (52.4)	18 (75)	8 (100)	12 (50)	4 (18.2)	1 (3.7)
Yes, for most residents	1 (5.9)	3 (11.1)	4 (19)	1 (4.2)	0 (0)	1 (4.2)	5 (22.7)	2 (7.4)
Yes, for some residents	2 (11.8)	4 (14.8)	1 (4.8)	1 (4.2)	0 (0)	4 (16.7)	1 (4.5)	4 (14.8)
No, never	6 (35.3)	10 (37)	5 (23.8)	4 (16.7)	0 (0)	7 (29.2)	12 (54.5)	20 (74.1)
**Specific guidelines used for the last 3 days of life of a resident in need of palliative care in your facility?**								
Yes	8 (47.1)	10 (37)	12 (52.2)	21 (51.2)	2 (28.6)	1 (4.2)	2 (9.1)	5 (20)

Due to convergence problems because of low numbers of cases the significance of these differences could not be tested

^a^Nursing home type: In this table Belgium and Finland are not reported since they only have type 2 homes. Type 1 includes nursing homes with 24/7 on-site physicians, nurses and care assistants, type 2 are nursing homes with 24/7 on-site nurses and care assistants and off-site physicians and type 3 consists of nursing homes with 24/7 on-site care assistants and off-site nurses and physicians

## Abbreviations

BANS-S: The Bedford Alzheimer Nursing Severity Scale
GDS: The Global Deterioration Scale
CPS: The Cognitive Performance Scale
PACE: Palliative Care for Older People in Europe
IMPACT: IMplementation of quality indicators for PAlliative Care sTudy
ENRICH: ENabling Research In Care Homes
ATOME: Access To Opioid Medication in Europe

## References

[1] Singh AA, Boyle JR (2020). Evaluating quality in clinical care. Surg Oxf Oxfs.

[2] van Riet PJ, Vernooij-Dassen M, Dröes RM, Radbruch L, Vissers K, Engels Y (2014). Consensus on quality indicators to assess the organisation of palliative cancer and dementia care applicable across national healthcare systems and selected by international experts. BMC Health Serv Res.

[3] Castle NG, Ferguson JC (2010). What is nursing home quality and how is it measured?. Gerontologist.

[4] Iliffe S, Davies N, Manthorpe J, Crome P, Ahmedzai SH, Vernooij-Dassen M (2016). Improving palliative care in selected settings in England using quality indicators: a realist evaluation. BMC Palliat Care.

[5] Woitha K, Hasselaar J, van Beek K, Ahmed N, Jaspers B, Hendriks JC (2015). Testing feasibility and reliability of a set of quality indicators to evaluate the organization of palliative care across Europe: a pilot study in 25 countries. Palliat Med.

[6] Froggatt K, Arrue B, Edwards M (2017). Mapping palliative care systems in long term care facilities in Europe: report of an EAPC Taskforce.

[7] Van den Block L, Smets T, van Dop N, Adang E, Andreasen P, Collingridge Moore D (2016). Comparing palliative care in care homes across Europe (PACE): protocol of a cross-sectional study of deceased residents in 6 EU countries. J Am Med Dir Assoc.

[8] Research Ready Care Home Network | ENRICH. Available from: http://www.enrich.nihr.ac.uk/pages/research-ready-care-home-network. Cited 2019 May 22.

[9] Froggatt K, Reitinger E (2013). Palliative care in term care settings for older people. Report of an EAPC Taskforce 2010–2012.

[10] Honinx E, van Dop N, Smets T, Deliens L, Van Den Noortgate N, Froggatt K (2019). Dying in long-term care facilities in Europe: the PACE epidemiological study of deceased residents in six countries. BMC Public Health.

[11] van Riet PJ, Engels Y, Iliffe S, Radbruch L, Kaasa S, Chattat R (2014). Improving the organization of palliative care by implementing quality indicators and national and setting-specific interventions: study protocol of the IMPACT project. Prog Palliat Care.

[12] IBM Corp. IBM SPSS Statistics for Macintosh, Version 23.0. Armonk: IBM Corp; 2015.

[13] Pivodic L, Smets T, Van den Noortgate N, Onwuteaka-Philipsen BD, Engels Y, Szczerbińska K (2018). Quality of dying and quality of end-of-life care of nursing home residents in six countries: an epidemiological study. Palliat Med.

[14] Wulf JD, ZorgnetIcuro (2020). Woonzorgcentra in Vlaanderen.

[15] Payne S. Palliative care nursing: principles and evidence for practice. McGraw-Hill Education (England); 2008. p. 733.

[16] Lindqvist O, Lundquist G, Dickman A, Bükki J, Lunder U, Hagelin CL (2013). Four essential drugs needed for quality care of the dying: a Delphi-study based international expert consensus opinion. J Palliat Med.

[17] Carroll T, Quill T. Use of generalist and specialist palliative care for older people. Palliative care for older people. Oxford University Press. Available from: https://oxford.universitypressscholarship.com/view/10.1093/acprof:oso/9780198717614.001.0001/acprof-9780198717614-chapter-17. Cited 2021 Feb 11.

[18] Toscani F, Di Giulio P, Villani D, Giunco F, Brunelli C, Gentile S (2013). Treatments and prescriptions in advanced dementia patients residing in long-term care institutions and at home. J Palliat Med.

[19] Duthey B, Scholten W (2014). Adequacy of opioid analgesic consumption at country, global, and regional levels in 2010, its relationship with development level, and changes compared with 2006. J Pain Symptom Manage.

[20] Neumann-Podczaska A, Nowak T, Suwalska A, Łojko D, Krzymińska-Siemaszko R, Kozak-Szkopek E (2016). Analgesic use among nursing homes residents, with and without dementia, in Poland. Clin Interv Aging.

[21] De Lima L, Pastrana T, Radbruch L, Wenk R (2014). Cross-sectional pilot study to monitor the availability, dispensed prices, and affordability of opioids around the globe. J Pain Symptom Manage.

[22] Tanghe M, Van Den Noortgate N, Pivodic L, Deliens L, Onwuteaka-Philipsen B, Szczerbinska K (2019). Opioid, antipsychotic and hypnotic use in end of life in long-term care facilities in six European countries: results of PACE. Eur J Public Health.

[23] Tanghe M, Van Den Noortgate N, Deliens L, Smets T, Onwuteaka-Philipsen B, Szczerbińska K (2020). Opioid underuse in terminal care of long-term care facility residents with pain and/or dyspnoea: a cross-sectional PACE-survey in six European countries. Palliat Med.

[24] Hendriks SA, Smalbrugge M, Hertogh CMPM, van der Steen JT (2014). Dying with dementia: symptoms, treatment, and quality of life in the last week of life. J Pain Symptom Manage.

[25] Morgan MM, Christie MJ (2011). Analysis of opioid efficacy, tolerance, addiction and dependence from cell culture to human. Br J Pharmacol.

[26] Graczyk M, Borkowska A, Krajnik M (2018). Why patients are afraid of opioid analgesics: a study on opioid perception in patients with chronic pain. Pol Arch Intern Med.

[27] Final Report Summary - ATOME (Access to Opioid Medication in Europe) | Report Summary | ATOME | FP7 | CORDIS | European Commission. Available from: https://cordis.europa.eu/project/id/222994/reporting/de. Cited 2020 Apr 28.

[28] Vranken MJ, Linge-Dahl L, Mantel-Teeuwisse AK, Radbruch L, Schutjens MHD, Scholten W (2020). The perception of barriers concerning opioid medicines: a survey examining differences between policy makers, healthcare professionals and other stakeholders. Palliat Med.

[29] Froggatt K, Payne S, Morbey H, Edwards M, Finne-Soveri H, Gambassi G (2017). Palliative care development in European care homes and nursing homes: application of a typology of implementation. J Am Med Dir Assoc.

[30] Honinx E, Van den Block L, Piers R, Van Kuijk SMJ, Onwuteaka-Philipsen BD, Payne SA, Szczerbińska K, Gambassi GG, Finne-Soveri H, Deliens L, Smets T. PACE. Potentially Inappropriate Treatments at the End of Life in Nursing Home Residents: Findings From the PACE Cross-Sectional Study in Six European Countries. J Pain Symptom Manage. 2021;61(4):732–42.e1. 10.1016/j.jpainsymman.2020.09.001. 10.1016/j.jpainsymman.2020.09.001 32916262

[31] Reitinger E, Froggatt K, Brazil K, Heimerl K, Hockley J, Kunz R (2013). Palliative care in long-term care settings for older people: findings from an EAPC Taskforce. Eur J Palliat Care.

[32] Arias-Casais N, López-Fidalgo J, Garralda E, Pons JJ, Rhee JY, Lukas R, et al. Trends analysis of specialized palliative care services in 51 countries of the WHO European region in the last 14 years: Palliat Med. 2020. Available from: https://journals.sagepub.com/doi/full/10.1177/0269216320931341. Cited 2020 Jun 18. 10.1177/0269216320931341 PMC7388149 32519584

[33] Woitha K, Garralda E, Martin-Moreno JM, Clark D, Centeno C (2016). Ranking of palliative care development in the countries of the European Union. J Pain Symptom Manage.

[34] Spasova S, Baeten R, Coster S, Ghailani D, Peña-Casas R, Vanhercke B, et al. Challenges in long-term care in Europe: a study of national policies 2018. 2018. Available from: http://dx.publications.europa.eu/10.2767/84573. Cited 2020 Jun 18.

